# Effect of intensive nutrition education and counseling on hemoglobin level of pregnant women in East Shoa zone, Ethiopia: randomized controlled trial

**DOI:** 10.1186/s12884-023-05992-w

**Published:** 2023-09-19

**Authors:** Ermias Bekele Wakwoya, Tefera Belachew, Tsinuel Girma

**Affiliations:** 1 Department of Nutrition and Dietetics, Jimma University, P.O.Box 378, Jimma, Ethiopia; 2 2Departments of Pediatrics and Child Health, Jimma University, Jimma, Ethiopia

**Keywords:** Ethiopia, Hemoglobin, Nutrition, Pregnancy, RCT

## Abstract

**Background:**

The aim of this study was to assess the effect of intensive nutrition education and counseling on hemoglobin level during pregnancy.

**Methods and materials:**

The study was a one year two-arm parallel design cluster randomized controlled trial in East Shoa zone, Ethiopia. End-line data were collected from 163 intervention and 163 control group pregnant women. The intervention was a three consecutive trimester based counseling sessions using health belief model, weekly regular SMS sent on mobile phone containing core message and providing leaflet with food menu of Iron rich diet. The women in the control group received routine nutrition education from facilities. After adjusting for potential confounders, a linear mixed-effects model was used to assess the intervention effect.

**Results:**

There was a significant change in both hemoglobin level and proportion of anemia in the intervention group. The mean hemoglobin level within intervention group before and after intervention was (12.08± 1.15, 12.53± 1.18) with *p* value of 0.01. The prevalence of anemia among intervention group declined from 14.7 % at the baseline to 9.2% after intervention. At the end of the trial, women in the intervention group had significantly better hemoglobin level than women in the control group (β = 0.50, *p <* 0.01).

**Conclusion:**

The intervention was effective in improving the hemoglobin level and consumption of iron rich diet among pregnant women. Therefore, employing trimester based counseling by using HBM constructs and regular reminding messages have to be provided to pregnant women as part of the regular antenatal care service.

**Supplementary Information:**

The online version contains supplementary material available at 10.1186/s12884-023-05992-w.

## Introduction

Anemia is a condition in which the hemoglobin concentration required to carry oxygen in venous blood is < 11 g/dl in the first and third trimesters and 10.5 g/dl in the second trimester [1, 2]. Anemia in pregnancy is one of a serious global public health concern with the burden falling on low and middle income countries [3].

The prevalence of anemia among pregnant women is estimated to be 38% worldwide, 36.9% in Africa and 23% in Ethiopia [4, 5]. Anemia is classified in to mild, moderate and severe. The Hgb level for each class of anemia during pregnancy are 10.0–10.9 g/dL (mild), 7–9.9 g/dL (moderate) and < 7 g/dL (severe) [5].

Anemia can be caused by both nutritional and non-nutritional factors, with iron deficiency being the most common cause [6]. Intestinal parasite, poor nutritional intake, repeated infections, frequent pregnancies and low health-seeking behaviors are associated with anemia [7, 8].

Globally, Anemia is estimated to contribute for more than 115,000 or 20% of all maternal deaths and is also responsible for 591,000 prenatal deaths per year [9, 10]. Pregnant women suffering from anemia and their neonates encounter negative consequences, including experience of general fatigue, fetal anemia, preterm delivery, low birth weight, increase risk of postpartum hemorrhage, intrauterine growth restriction, perinatal mortality, stillbirth, reduced work capacity, low tolerance to infections, shortness of breath, reduced physical and mental performance [11, 12].

Anemia can be prevented by creating awareness for pregnant mothers on iron supplementation, counseling on taking iron reach diet, deworming, consistent use of insecticide treated bed net, nutritional counseling, food diversification, iron and folic acid supplementations and by treating the underlying causes and complications [13, 14]. The mostly used strategy to improve nutritional status of pregnant women is nutrition education that emphasizes on maternal diet quality [15].

One of In Ethiopia, the routine nutrition education given for pregnant women by the health system are inconsistent and mostly the nutritional counselling is also missed most of the time. Even in the healthcare institutions where the nutritional counselling is provided to pregnant women, it is incomplete and not supported with appropriate visual aids. Health care professionals has been advising pregnant women to eat one additional meal from available foodstuffs [16]. Therefore, appropriate counseling on the maternal diet before and during pregnancy seems to be of high priority to promote positive pregnancy outcomes [17].

In Ethiopia, data on the effect of intensive nutrition education and counseling intervention on hemoglobin level of pregnant women were scarce. Thus, the aim of this study was to assess the effect of intensive nutrition education and counseling in improving the hemoglobin level of pregnant women in East Shoa Zone. The results of the study will be an input to planners and policymakers at the national and regional level to amend nutrition education during pregnancy.

## Methods and materials

### Study setting and period

The study was conducted in East Shoa Zone from January 4, 2021 to February 28, 2022. The zone is one of the 20 zones in the Oromia Region. In Ethiopia, a region and zone are the first and second level administrative divisions, respectively. Zones are further divided into a number of woredas. The total population in the East Shoa zone is 1,567,953 of which 48.3% were female. The number of estimated pregnant women was 54,408. According to the East Shoa zone health office, the health service delivery is organized under 3 hospitals, 59 health centers, and 290 health posts. The food production system in the district is characterized by mixed crop-livestock farming.

### Trial design

The study was a one year two-arm parallel design cluster randomized controlled trial, conducted with the principles of Helsinki Declaration and the requirements of Good Clinical Practice [18].

### Inclusion and exclusion criteria

Pregnant women were identified using urine pregnancy test. The study enrolled pregnant women before 16weeks of gestational age. Pregnant women are recruited at early stage of their pregnancy to effectively observe the effect of NEC on their hemoglobin level. Pregnant women who had chronic illness such as hypertension, diabetic mellitus and mental illness were excluded from the study. Additionally, Women who refused to give verbal consent and who had intention of leaving the study area until delivery were excluded.

### Sample size determination

The sample size was calculated using G power 3.1.9.2 with an assumptions of a precision of 5%, power of 85%, effect size of 0.5 and mean hemoglobin change of 0.8gm/dl over the study period [19, 20].

Since cluster sampling was used, the calculated sample size was multiplied by design effect of two. Considering a 10% non-response rate and 20% loss through follow up and drop out, the final sample size was 224 pregnant women in both arms. Therefore, 366 women who fulfilled the requirement were enrolled to the trial in intervention and control arm.

### Randomization of clusters and allocation concealment

On the basis of the health map, East Shoa zone has 10 rural woredas comprising of 159 health centers. Using a random number table generated in Excel software, a data manager, who was not member of the research team, randomly allocated three woredas for the intervention arm and the other three for the control arm, and then randomly selected one health center per woreda as clusters for the study. The non-adjacent catchment area health centers (clusters) selected were randomly allocated to the intervention and control arm by 1:1 ratio.

Enrollment in the study was proposed to all eligible pregnant women attending antenatal visits during the recruitment period in the selected health facilities Systematic random sampling techniques were used to select participants by following K^th^ value. The Kth value was calculated by taking the total number of pregnant women on ANC during the study period and dividing it for the sample size and it was found to be six. The first comer was considered as the first participants and participants who came at the 6th interval were included until the determined sample size was achieved.

Cluster randomization was used to prevent message contamination. Buffer zones (non-adjusted catchment area health centers) were also left between the intervention and control clusters to prevent information contamination. The intervention was blinded to pregnant women, counselor and data collector.

### Intervention

Participants were either assigned to control or intervention group. Pregnant women assigned to control group received routine health education given by health care system and pregnant women in in intervention group received a package of intensive nutrition education and counseling (INECP) which contains 3 components. The intervention includes; intensive nutrition counseling given using health believe model by trained midwives in three sessions, weekly serial SMS(short message service) text delivered on mobile phone using local languages. Each pregnant woman has received a total of 18 SMS during the study period. Take home brochures prepared in local languages were distributed to pregnant women at intervention health centers.

A three-day intensive training with role-playing was given to the counselors and supervisors using the training manual. The training module was modified from blended and integrated nutrition learning module (BINLM). The core message was adapted from World Health Organization and Ethiopian Ministry of Health national nutrition module.

Six BSc midwives were recruited as counselors and supervisors of the counseling process respectively. The training was facilitated by the principal investigator. Invited nutritionists who has certified by training of trainers (TOT) in BINLM supervised the training process. The nutrition education and counseling were provided based on the HBM and the information were framed in a way that addresses all HBM constructs like Perceived susceptibility(the belief about the likelihood of developing a health problem or experiencing negative outcomes),Perceived severity(the assessment of the seriousness or consequences of a health problem),Perceived benefits (the belief about the positive effects of taking action to prevent or treat a health problem), Perceived barriers (the belief about the costs or obstacles of taking action), Cues to action (the factors that trigger or motivate action, such as symptoms, media messages, or social influences), Self-efficacy (the confidence in one's ability to perform a health behaviour successfully). Health belief model was selected because, the desired behavioral change is at an individual level and due to its convenience and simplicity.

The training had a brief lecture and explanation on nutritional education and counselling intervention during pregnancy which includes; Promoting a healthy eating habits by increasing the diversity and amount of foods consumed with increasing gestational age, giving emphasis on iron reach meal, promoting adequate sufficient protein and energy intake, promoting consistent and continued use of iron folic acid supplements, use of iodized salt, counseling about healthy eating and reducing heavy workload, preventive deworming after the first trimester and impregnated bed net use and utilization of health care services.

Moreover, susceptibility, severity and consequences of malnutrition to pregnant women and her fetus were also discussed during counseling. The benefits of taking an adequate amount of diversified meals and barriers that interfere with taking a balanced diet and developing self-confidence to follow the right dietary practice were also included in the counseling guide. Additionally, the training was aimed to improve important counseling skills and GALIDRA steps (Greet, Ask, Listen, Identify, Discuss, Repeat, and Appoint) was discussed in detail. The training session had a brief discussion on using health belief model in nutrition education and counseling as a delivering modality of messages and recommendations.

The training was supported by power point presentations, discussions and role plays. Trainees were also provided additional materials such as modules and summary pamphlets. The training was evaluated by the results of Pre-post test questions given to the participants. A close follow up was made on counseling process by supervisors and identified challenges and gaps were solved.

Each pregnant woman attended three counseling sessions throughout her pregnancy period. Individual nutrition counseling was given by trained midwives. The first counselling session was before 16 weeks, second session 24 – 28 weeks and the third session were after 28 weeks of gestation. During counseling, counselors used a client-centered approach to identify pregnant women’s dietary practices and their specific needs in terms of nutrition. Counselors considered women’s needs, household income and identified gaps and allowed the women to choose recommendations that were locally available, acceptable and affordable.

Counseling was delivered monthly using a counseling guide with the core contents and each counseling session lasts for 30-45 minutes. The first counseling was given before 16weeks of gestation, focused on basic nutrition, food groups, food selection, preparation, meal frequency, portion size, and iodized salt utilization. The second sessions of the counseling were given during the second trimester of pregnancy and covered the whole contents of the counseling guide. The last counseling was given based on the identified gaps during the early third trimester of pregnancy. Module, pamphlets, MUAC tape and revised ANC logbook was provided to health centers in intervention group.

Take home brochures prepared in local languages (Afan Oromo and Amharic) was distributed to pregnant women attending intervention health facilities. The brochures include simple and easy to understand key messages and doable actions of maternal recommendations as bullet points and explanations of components of pregnancy nutrition. Messages of the leaflet and serial short SMS text prepared in local language delivered to intervention group also incorporated health belief model as a way of delivering the message.

### Outcome measures

The primary outcome was the effect of nutrition education and counseling intervention on a change in the hemoglobin level which was measured before 16weeks and 35-37weks. The secondary outcome of the study was gestational weight gain and dietary Iron intake. The difference in difference measure of the intervention group compared with the difference in the control group.

### Follow-up protocol

Pregnant women were scheduled for their subsequent ANC follow-up after the baseline data collection. Each woman's contact information was gathered when collecting baseline data. Each pregnant woman's home and mobile phone numbers, as well as those of her husband and any neighbors or friends, were collected. Using their phone number, pregnant women were located and notified of the date of their upcoming ANC visit. For all pregnant women in the intervention arm, a three-page home-use leaflet with a core message containing iron-rich diet menu prepared in local languages (Afan oromo and Amharic) was given. Pregnant women in the intervention arm received 18 weekly short SMS texts prepared in the local language delivered via mobile phone device.

Data collectors reminded a pregnant woman 3 days before appointment for next visit and call again on her day of appointment. Husbands and home contacts were used for women whose cellphone numbers weren't functional. If those numbers could not be reached, a pregnant woman may be located using the contact information of a friend or neighbor. In cases where a lady doesn't answer the phone or has an unreachable phone number, data collectors made a second attempt and repeated calls for three days in a row. The woman was called three days before to her visit and on the day of her appointment in a process that was repeated before end line data collection. Data collectors were on standby at the ANC clinic and pregnant women were reminded of their follow-up appointment during the phone calls. After a significant effort at persuasion on the part of the data collectors, small transportation incentives were provided to pregnant women who were unable to attend their end-line appointments due to financial difficulties.

Lost to follow-up was declared when all these methods of tracing a woman failed, a woman changed health facility to different arm and who was referred to hospitals for various reasons, pregnant woman moves from the study area and who couldn’t return during the study period, The above follow up protocol was applied for both intervention and control health facilities the same way and independently.

### Data collection tools and measurement

Data was collected through a pretested and structured interviewer-administered questioner, anthropometric measurement and a sample collection for hemoglobin measurement form January, 2021 to February 2022. The questionnaires included socio-economic characteristics of women and their households, dietary habit and food intake, food frequency questions (FFQ), obstetric history, prenatal health seeking behavior, and measure variables relevant to maternal nutrition during pregnancy. The questionnaire developed after a thorough review of literature on the subject matter. Data on socio-demographic characteristics and obstetric history were collected at the baseline. Whereas, data on hemoglobin level measurement, food security, nutrition knowledge were taken before and after implementation of the intervention.

Six data collector midwives, 4 medical laboratory technologist and 2 supervisors were trained for 3 days using a training manual focused on data collection tool and procedure. Questionnaires initially written in English and translated to Afan Oromo and Amharic then back translated to English by an expert fluent in English and the local languages. The tools were pre-tested before using for actual data collection and questionnaires’ consistency reliability test was done and a Cronbach’s alpha coefficient of >0.70 was held to indicate reliability. The knowledge questionnaire consisted of definitions, causes, signs and symptoms, effects of anemia on mother and child health, and methods of preventing anemia. It was developed according to literature review [21]. A correct answer was scored as “one” and an unknown or incorrect answer was scored as “zero.” The total possible score ranged from 0 to 20 points, with higher scores reflecting higher levels of knowledge. The cronbach’s alpha for the knowledge questionnaire was 0.76.

All data were obtained in the respondents’ mother tongue language. The privacy of the women was secured during the interview. The supervisors and the principal investigator monitored the data collection procedure. The data collection and counselors team held a daily meeting to discuss challenges encountered during the day. The food frequency questioner was used to find the frequency of iron-rich food intake over the past months. We ranked the frequency of intake on a seven-level scale: 7 indicated a frequency of once a day, 6 of 4-6days per week,5 of 2-3 times per week,4 of once per week, 3 of 2-3 times per month,2 of once per month, 1 of never eat. The FFQ consisted of 121 foods selected form Ethiopian food composition table and only 81 foods were reliable enough to be included in the final version of the questionnaire with cronbach’s alpha coefficient of 0.7.The total possible score ranged from 122 to 274 points; higher scores reflected higher frequencies of intake.

Hemoglobin was measured using a portable and battery-operated machine (HemoCue, Angel Holm, Sweden). After swiping the site with disinfectant, a finger prick was made to obtain blood for hemoglobin measurement. The first two drops were swapped away and the third drop was used to fill the micro cuvette for reading of the hemoglobin. The hemocue machine had a sensitivity of 85% and specificity of 94% for capillary blood [22]. The primary outcome of the study was hemoglobin level of the pregnant women that was measured before and after intervention. Post-intervention data were measured from 36 to 38weeks of pregnancy. Pregnant women who didn’t attend all counseling sessions were considered as ‘did not adhere to the guideline’ and those withdraw from participating in the study were taken as ‘lost to follow up’.

### Intervention fidelity

Based on the Health Behavioral Change Consortium recommendations the intervention given was assessed by fidelity criteria checklist [23]. The checklist includes intervention design assessment, counseling process, training of counselors, and receipt of intervention and enactment of skills gained from the intervention [24].

The study took equal numbers of clusters for intervention and control groups. Non-adjacent clusters (health centers) were selected to prevent information contamination. The trial used a control group and counseling guide. The intervention process was pretested before the implementation of the trial. Each woman in intervention arm received equal number, length of contact and frequencies of counseling session.

A Counseling skill training was given using training manual, and it includes a role-play and mock counseling practice. The training session has pre and post-training tests and practical evaluation. Process evaluation of the counseling session was done for randomly selected sessions by process evaluator. Participants, counselors, and data collectors were blinded to the study hypotheses. Additionally, the data entry process was blinded by labeling the data with a unique number until analysis was finalized. The counseling process was supervised by principal investigator and supervisors. Intervention receipt was assessed by awareness of the women on diet during pregnancy through interviewing about their understanding of the core contents of the intervention.

### Data analysis

The data were coded and entered into epidata V.3.1 to minimize design skipping patterns and logical errors. A cleaned copy of data was transported from epidata to SPSS version 26 for data analysis. Descriptive statistics were used to summarize the baseline socio-demographic characteristics of the women by group status. A chi-square test was performed to compare the baseline characteristics of the intervention and control groups. Principal component analysis was used to construct the wealth index. Then the wealth index was classified into wealth quintiles. The procedure of the wealth index is described elsewhere [21].

Comparisons of hemoglobin level between and within the control and intervention groups were done using independent samples and paired sample t-tests, respectively. In all analysis, A two-sided *p*-value of < 0.05 was used as a cutoff point to declare statistical significance. Multicollinearity between the independent variables was assessed by using variance inflation factors (VIF >10 was considered as existence of collinearity).

A per-protocol analysis was performed in this study. Pregnant women who attended three counseling session and gave end line data were included in the final analysis. A linear mixed-effects model was used to determine the effect of the intervention on changes in the hemoglobin level of pregnant women over time. This model enables to accommodate the correlation of observations due to the repeated measures (pre- and post- intervention) and the clustering of individuals within the 6 randomly selected clusters.

Before fitting the model, the normality assumption of the outcome variable, hemoglobin level, was tested using Shapiro-Wilk’s test, and the test revealed that the assumption was satisfied (p > 0.05). We used the Akaike information criterion (AIC) to help us choose the best statistical model. We selected the model that had the smallest AIC. During fitting the model, participants and clusters were analyzed as random effects.

The model also enables to control the effects of potential confounding factors (food security, wealth index, education, family size, age).Variables in the bivariate linear mixed regression model with *p*-values less than 0.2 were selected as candidate variables for the multivariable linear mixed model analysis. Therefore, the two-level model was fitted to account for time-invariant variables at the individual level. The effect of the intervention was evaluated by testing the interaction term between time and treatment allocation. All statistical analyses were performed using the SPSS package version 26.

## Result

### Socio- demographic characteristics

A total of 366 eligible pregnant women were recruited to the study from six health centers. Of which, 326 women (163 from each arm) who were strictly adhered to the protocol were included in the final analysis. Baseline data on age, ethnicity, wealth index, occupation, family size, religion and educational status of the respondents were similar between the two arms (*P* > 0.05).

The age of the pregnant women enrolled in the study ranges between 18-45 years (mean ± SD =25.8± 4.49).The study participants had an average family size of 3.07(±1.11) and 4.04(±4.56) in intervention and control group respectively. The socio-demographic characteristics of the study participants are available elsewhere [21].

### Response rate and attendance

**Fig. 1:**
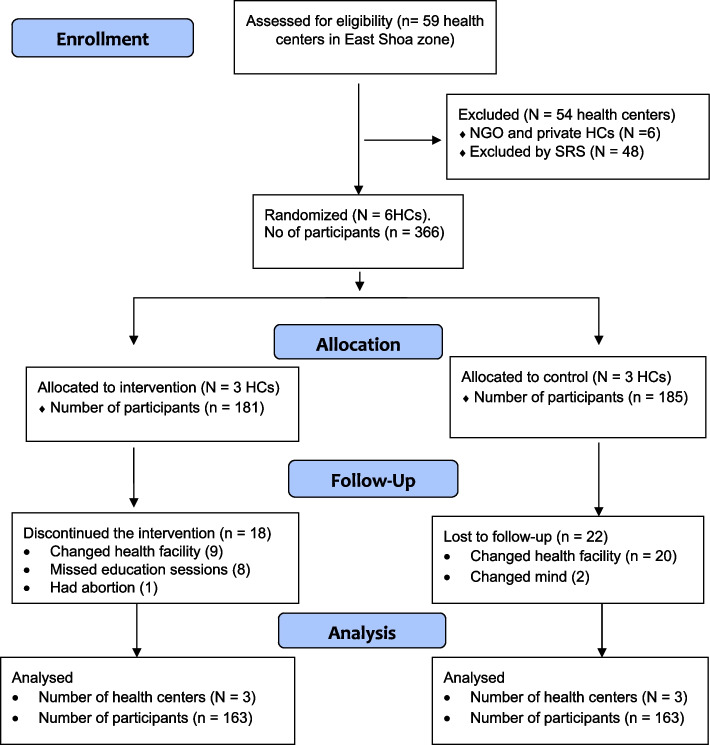
The flow of the study participants through the trial according to the criteria recommended in the CONSORT guideline. HCs – Health Centers, NGO – Non Governmental Organization, SRS – Simple Random Sampling

A total of 366 participants were surveyed at the base line, of which 326 women who were strictly adhered to the protocol were included in the final analysis with 89.1% retention rate. All participants in the intervention and control group attended the first session. 18(9.9%) from intervention group discontinued the intervention because of missed education session [8], changed health facility [9], had abortion [1] and a total of 22(11.8%) from control group lost from the follow up. The overall follow up of study participants through the trial was summarized by CONSORT guideline, flow chart (Fig. 1).

### Gestational weight gain of pregnant women

The present study shows average maternal weight gain was higher in the intervention group compared to control group (9.69 kg vs. 7.75kg) (Table 1). There was no significant difference in gestational weight gain of pregnant women between the groups post intervention (Table 2).

**Table 1:** Comparison of gestational weight gain between control and intervention groups among pregnant women in East Shoa zone

Variables	Control group(n 163)	Intervention (n 163	*P*-value
	Mean ± SD	Mean ± SD	
Weight(kg)			
Baseline	60.49(8.23)	59.53(8.0)	0.28
End line	68.24(8.15)	69.22(8.01)	0.27
Change	7.75(2.02)	9.69(3.54)	≤ 0.01

**Table 2:** Mean gestational weight gain difference of the differences between intervention and control group among pregnant women attending ANC service in East Shoa zone, central Ethiopia

Variable	Mean difference (EL-BS) intervention group	Mean difference (EL-BS) control group	Mean difference (Edline – Baseline) (± SD)	*P* value
Mean Gestational weight gain	9.69(3.54)	7.75(2.02)	1.93	0.12

### Dietary intake of iron rich diet among intervention and control group

At baseline, there was no statistically significant difference in the consumption of an iron-rich diet between the two groups.

At the End of the trial, the intervention group consumed considerably more dark green veggies, vitamin C-rich diet, dairy products, eggs, and red meat than the control group did (Table 3)

**Table 3:** Comparison of dietary intake of Iron rich food items between control and intervention groups using seven days food frequency questionnaire

**Variables**	**Control group(*n* = 163)**	**Intervention group(*n* = 163)**	
**Food groups**	**Mean ± SD**	**Mean ± SD**	***p* value**^**1**^
**Red meat, liver, fish**			
Base line	17.6 ± 4.04	17.39 ± 4.16	0.85
End line	18.06 ± 3.36	22.66 ± 4.77	< 0.001^*^
Change	0.46 ± 0.53	5.27 ± 0.54	4.81(< 0.01)
**Vitamin C rich diet**			
Base line	4.35 ± 2.11	4.20 ± 2.14	0.729
End line	4.20 ± 2.26	5.42 ± 2.01	0.005^*^
Change	0.04 ± 2.85	1.41 ± 2.07	1.37(< 0.01)
**Legumes and nuts**			
Base line	14.65 ± 2.95	14.58 ± 3.06	0.86
End line	16.47 ± 3.9	21.65 ± 5.05	< 0.01
Change	1.82 ± 4.16	7.07 ± 5.87	5.25(< 0.01)
**Grains/ Cereal/**			
Base line	37.82 ± 6.76	37.391** ± **6.85	0.63
End line	40.65 ± 6.26	46.77 ± 7.73	< 0.01
Change	2.8 ± 0.82	9.39 ± 0.91	6.59(< 0.01)
**Eggs**			
Base line	3.34 ± 1.29	3.18 ± 1.27	0.33
End line	3.35 ± 1.1	4.02 ± 1.04	< 0.01
Change	0.01 ± 0.59	0.83 ± 1.28	0.82(< 0.01)
**Dark green leafy vegetables**			
Base line	9.08 ± 3.03	8.86 ± 3.19	0.56
End line	10.37 ± 2.27	17.96 ± 4.23	< 0.001^*^
Change	1.27 ± 3.04	9.11 ± 5.21	7.84(< 0.01)

^**1**^Independent sample t-test

^*^Statistical significance is at p 0.05

### Hemoglobin status of study participants

Prior the implementation of the intervention package there was no significant difference in the proportion of anemia among of pregnant women enrolled in the intervention and control groups. (0.14 Vs 0.17) with *p* value *p=* 0.57 (Table 4). Similarly, the mean hemoglobin level between intervention and control group at baseline were not statistically significant (12.08±1.15, 12.21±1.44) with *p* value of 0.37.

**Table 4:** Mean difference between baseline and end line hemoglobin measurement among pregnant women attending ANC service in East Shoa zone, central Ethiopia (paired t test)

Type of groups	Baseline mean hemoglobin(± SD)	Endline mean Hemoglobin(± SD)	Mean difference (Edline – Baseline) (± SD)	*P* value
Intervention groups	12.08(1.15)	12.53(1.18)	0.45(1.57)	0.01
Control group	12.2(1.44)	12.19(1.16)	-0.01(1.81)	0.95

There was a significant change in both hemoglobin level and proportion of anemia between intervention and control group after the intervention was implemented. The mean hemoglobin level within intervention group before and after intervention is (12.08± 1.15, 12.53± 1.18) with *p* value of 0.01 (Table 4). The prevalence of anemia among intervention group declined from 14.7 % at the baseline to 9.2% after intervention. The result showed that the intervention improves the mean hemoglobin level by 0.45 g/dL (Table 5).

**Table 5:** Mean hemoglobin difference of the differences between intervention and control measurement among pregnant women attending ANC service in East Shoa zone, central Ethiopia (independent t test)

Variable	Mean difference (EL-BS) intervention group	Mean difference (EL-BS) control group	Mean difference (Edline – Baseline) (± SD)	*P* value
Mean hemoglobin	0.45(1.57)	-0.01(1.81)	0.46	0.04

### Effect of nutrition education and counseling on hemoglobin level of pregnant women

The variability of average hemoglobin level across individual was 1.42 and statistically significant (*p <* 0.05). In this study since the intra individual correlation coefficient was 0.02, two level models were fitted (Table 6). After controlling for food security, family size, educational status, women decision making and wealth women in intervention group showed significantly improved in hemoglobin level at the end of the study trial (β=0.50, *p<*0.01).

**Table 6:** Linear mixed effect model predicting hemoglobin level of pregnant women in East Shoa zone, Central Ethiopia

Fixed effectVariables	Model 1		Model 2		Model 3	
	Estimate (SE)	95% CI	Estimate (SE)	95%CI	Estimate(SE)	95%CI
Intercepts	12.52(0.09)	12.34–12.7)	12.29(0.06)	12.15,12.43	24.68(0.66)	23.38, 25.97
Intervention effect			**0.51(0.04**)	0.43,0.60	**0.50(0.08)**	0.33, 0.67
Endline control			-0.09(0.07)	-0.05,0.25	**-0.06(0.09)**	-0.25,012
Baseline intervation			**0.08(0.08)**	-0.07,0.25	0.9(0.07)	0.98,1.01
Food security					0.01(0.27)	-0.54, 0.56
Educational status					-0.46(0.28)	-1.03, 0.08
Wealth					-0.46(0.45)	- 1.36, 0.43
Women decision making power					0.02(0.28)	- 0.53, 0.58
Family size					-0.19(0.45)	-1.08, 0.70
Nutrition knowledge						
Random effect						
Random effects	Model 1	Model 2	Model 3			
Variances	1.39(0.15)	1.17(0.18)	2.77(0.31)			
ICC	0.02	0.03	0.07			
AIC	520.84	258.9	656.21			
Parameters	3	6	14			

The intercept-only model estimates the variance of the cluster-level residual errors as 0.007 (variability of the average nutritional status across all clusters was 0.0035 and which wasn’t statistically significant (*p=*0.92). The intra-cluster correlation coefficient was closer to zero (0.002) which showed that no need for fitting a third- level model (Table 6).

## Discussion

This research provided further evidence that the prenatal nutrition education and counseling intervention improved hemoglobin levels and increased intake of iron-rich foods. The nutrition intervention given for pregnant women focus on dietary diversity, consumption of iron rich diet and diets which increase iron absorption, iron supplementation, and prevention of malaria and intestinal parasites, combined with appropriate antenatal care, significantly reduced the prevalence of anemia in pregnancy.

Similar result were observed by a quasi-experimental research conducted by Al-tell MA et al. (2010) found a substantial favorable correlation between dietary habits and raising pregnant women's hemoglobin levels. [25] .The counseling and continuous education intervention was only found to substantially increase hemoglobin change (0.23 gm/dl) when compared to the control group in a randomized control study among pregnant Nepalese women (P 0.01) [26].

Similar to this, individual education and counseling substantially increased the mean hemoglobin levels of pregnant women in a pre-test post-test research on pregnancy conducted by Garg & Kashyap (2006) (0.97 vs. 1.58, P0.001) [27]. A randomized research conducted at the University of Ghana found similar findings, demonstrating a favorable relationship between improved hemoglobin levels and nutrition instruction that emphasizes the intake of iron-rich foods (CG, 0.1+1.3 vs. -0.7+1.4) [28]. However, when compared to the control group, the intervention group's hemoglobin level had no meaningful impact, according to a randomized control study conducted in Greece [29].

The study also revealed that average gestational weight gain during pregnancy was higher in the intervention group compared to control group (9.69 kg vs. 7.75kg). Similar findings were reported by a quasi-experimental study conducted in Indonesia (2014), which showed that the treatment group experienced a higher average gestational weight gain than the control group(3.017kg vs. 1.80kg) [30]. Another study conducted by Kafatos AG et al, indicated that nutrition counseling during pregnancy improve dietary intake and maternal weight gain [29].

The current study's findings demonstrate that, compared to the control group, pregnant women who got nutrition instruction and a menu plan based on iron-rich foods consumed significantly more red meat, Legumes and nuts, Cereal/Grains, vitamin C-rich fruits, eggs and dark green veggies.

This outcome is consistent with a research from Dessie Town that found that pregnant women who received nutrition education and counseling using the HBM had significantly improved dietary habits [31]. Other studies have shown that nutrition instruction and guidance can enhance maternal diet, including dietary habits and nutrient intake [28, 32].

The success of this intervention may be attributed to the use of a counseling guide, serial weekly SMS messages sent on a mobile phone, and a nutrition message using a leaflet written in locally appropriate language with culturally appropriate pictures, which might help women in the intervention group remember key messages. Counseling was conducted using a trimester-based teaching technique and a client-centered, two-way dialogue approach. Counseling that encouraged behavior change towards having iron-rich food groups and a diversified diet is given based on the needs of specific women after assessing their existing socioeconomic situation, dietary practice, and knowledge of pregnancy.

Additionally, in this study, counseling was given using the HBM constructs. The finding of the study is in line with the study done in Northeast Ethiopia, which shows a positive result on improving pregnant women’s dietary practices by using TPB and HBM constructs during counseling [31].

This could be as a result of the fact that women who received counseling believed that anemia during pregnancy had serious negative impacts on both the mother and the fetus. This might be due to the fact that counseled women perceived that anemia during pregnancy had severe adverse effects on them and their fetus and also perceived themselves as vulnerable to the harmful effects of anemia, which motivated them to improve their dietary habits.

Implication: The results of this research have important practical repercussions for improving dietary counseling techniques, which will enhance maternal and infant health. The need for implementing the intervention package at health facility level through health workers using theory-enhanced methods is implied by the fact that considerably more pregnant women have adequate nutritional habits.

The merits of this research included its use of a client-centered approach and trimester-based counseling in a randomized controlled experiment. The study has some limitation as all responses were dependent on the women’s memory and honesty in answering questions and also due to the relatively short duration of the intervention, the post-intervention result might not have persisted longer.

## Conclusion

This study revealed that the intervention package containing consecutive counseling session using health believe model, weekly regular SMS sent on mobile phone containing core message and providing leaflet with food menu of Iron rich diet was effective in improving the hemoglobin level and consumption of iron rich diet among pregnant women. Therefore, employing trimester based counseling by using HBM constructs and regular reminding messages have to be provided to pregnant women as part of the regular antenatal care service. Furthermore, this research recommends developing a nutrition counseling guideline for ANC providers which include a thorough explanation of dietary recommendations during pregnancy and the HBM.

### Supplementary Information

**Additional file 1:** **Table S1.** Intervention Fidelity Strategies for Design of Study. **Table S2.** Intervention Fidelity Strategies for Monitoring and Improving Provider Training. **Table S3.** Intervention Fidelity Strategies for Monitoring and Improving Delivery of Intervention. **Table S4.** Intervention Fidelity Strategies for Monitoring and Improving Receipt of Treatment. **Table S5.** Intervention Fidelity Strategies for Monitoring and Improving Enactment of Treatment Skills.

## Data Availability

The raw data used for this study can be available upon request. Point of contact: Ermias Bekele Wakwoya. E-mail: ermi3577@gmail.com.
