# Supplementary material for: Effect of intensive nutrition education and counseling on hemoglobin level of pregnant women in East Shoa zone, Ethiopia: randomized controlled trial
Source: BMC Pregnancy Childbirth. 2023 Sep 19;23:676. doi: 10.1186/s12884-023-05992-w (PMC10507850; doi:10.1186/s12884-023-05992-w)
Supplement: Supplementary file 1 — Additional file 1: Table S1. Intervention Fidelity Strategies for Design of Study. Table S2. Intervention Fidelity Strategies for Monitoring and Improving Provider Training. Table S3. Intervention Fidelity Strategies for Monitoring and Improving Delivery of Intervention. Table S4. Intervention Fidelity Strategies for Monitoring and Improving Receipt of Treatment. Table S5. Intervention Fidelity Strategies for Monitoring and Improving Enactment of Treatment Skills. [file 12884_2023_5992_MOESM1_ESM.docx]

**Table S1: Intervention Fidelity Strategies for Design of Study**

| Goal | Description | Strategies |
| --- | --- | --- |
| Ensure the same treatment dose within conditions | -Ensure that treatment “dose” (measured by number, frequency, and length of contact) is adequately described and is the same for each subject within a particular treatment condition. | -Ensure fixed length, number, and frequency of contact sessions  -Ensure fixed duration of intervention protocol  -Record deviations from protocol regarding number, length, and frequency of contacts  -Ensure fixed amount of information for each treatment/control group  -Use treatment manual  -Monitor sessions and provide feedback to providers  -Training to providers |
| Ensure equivalent dose across conditions. | -Ensure that treatment dose is the same across conditions, particularly when conditions include multiple behavioral targets | -Have equal number of contacts for each intervention  -Use equal length of time for each intervention  -Use same level of informational content for each intervention. |
| Plan for implementation setbacks. | -Address possible setbacks in  implementation (e.g.,treatment  providers dropping out) | -Have pool of potential providers so that new providers need not be trained in a hurry  -Train extra providers beyond needed  -Have human backup  -Track provider attrition. |

**Table S2: Intervention Fidelity Strategies for Monitoring and Improving Provider Training**

| Goal | Description | Strategies |
| --- | --- | --- |
| Standardize training | -Ensure that training is conducted similarly  for different providers | -Ensure that providers meet a performance criteria  -Train providers together  -Use standardized training manuals/provider material/  -Have training take into account the different experience levels of providers  -Use structured practice and role-playing  -Observe intervention implementation with pilot participants  -Use same instructors for all providers  -Design training to allow for diverse implementation styles |
| Ensure provider skill acquisition | -Train providers to well-defined performance  criteria | -Observe intervention implementation with standardized patients  -Score provider adherence according to a prior checklist  -Conduct provider identified problem solving and debriefing  -Provide written exam pre- and post-training  -Certify interventionists |
| Minimize “drift” in provider skills | -Ensure that provider skills do not decay  over time (show that provider  skills demonstrated halfway through the  intervention period are not significantly  different than skills immediately after initial training) | -Conduct regular booster sessions  -Conduct in observation or recorded  -Encounters and review (score providers on their adherence using a priori checklist)  -Provide multiple training sessions  -Conduct weekly supervision or periodic meetings with providers  -Allow providers easy access to project staff for questions about the intervention  -Have providers complete self-report questionnaire  -Conduct patient exit interviews to assess whether certain treatment components were delivered. |
| Accommodate provider differences | -Ensure adequate level of training in providers of differing skill level, experience or professional background | -Have professional leaders supervise the team  -Monitor differential drop-out rates  -Evaluate differential effectiveness by professional experience  -Give all providers intensive training  -Use regular meetings  -Use provider centered training according to needs, background, or clinical experience  -Have inexperienced providers add to training by attending workshops or training programs |

**Table S3: Intervention Fidelity Strategies for Monitoring and Improving Delivery of Intervention**

| Goal | Description | Strategies |
| --- | --- | --- |
| Control for provider differences | -Monitor and control for subject perceptions of  nonspecific intervention effects | -Assess participants’ perception via self-report questioner  -Provide feedback to interventionist  -Select providers for specific characteristics  -Monitor participant complaints  -Have providers work with all treatment groups  -Have different supervisors |
| Reduce differences within treatment | -Ensure that providers in the same condition are delivering the same intervention. | -Use intervention protocol  -Provide a treatment manual  -Have supervisors rate audio and videotapes. |
| Ensure adherence to treatment protocol | -Ensure that the treatments are being delivered with regards to content and treatment dose | -Audio or videotape encounter and review with provider  -Randomly monitor audiotapes for both protocol adherence and nonspecific treatment effects  -Check for errors of omission and commission in intervention delivery  -After each encounter, have provider complete a behavioral checklist of intervention components delivered  -Ensure provider comfort in reporting deviations from treatment manual content |
| Minimize contamination between conditions | -Minimize contamination across treatment/ control | -Randomize sites rather than individuals  -Use treatment-specific handouts  -Use presentation materials/manuals  -Train providers to criterion with role-playing  -Give specific training to providers regarding the rationale for keeping conditions separate  -Supervise providers frequently |

**Table S4: Intervention Fidelity Strategies for Monitoring and Improving Receipt of Treatment**

| Goal | Description | Strategies |
| --- | --- | --- |
| Ensure participant comprehension | -Ensure that participants understand the information provided in intervention, especially when participants may be cognitively compromised, have a low level of literacy/education | -Use pre and post test process and knowledge measures  -Have providers ask questions/discuss material with subjects  -Complete activity logs; structure intervention around achievement-based on objectives  -Conduct structured interview with participants  -Have providers work with subjects until they can demonstrate the skills  -Have providers monitor and give feedback on practice sessions. |
| Ensure participant ability to use cognitive  skills | -Make sure that participants are able to use the cognitive skills taught in the intervention (e.g., reframing,  problem solving, preparing for high-risk situations | -Conduct structured interviews with participants  -Have providers work with participants until they can demonstrate skills  -Use measures of mediating variables  -Have providers monitor and give feedback on practice sessions  -Measure participant performance and completion of  training assignments  -Assess providers cognitive skills  -Let participants provide feedback  -Use questionnaires  -Use problem-solving structured interview that sets up hypothetical situations  -Ask participants to provide strategies for overcoming obstacles to changing their behaviors |
| Ensure participant ability to perform behavioral  skill | -Make sure that participants are able to use the behavioral skills taught in the intervention | -Observe subjects; use behavioral outcome measures  -Complete training assignments  -Monitor behavioral adherence  -Follow-up telephone contacts/counseling |

**Table S5:** **Intervention Fidelity Strategies for Monitoring and Improving Enactment of Treatment Skills**

| Goal | Description | Strategies |
| --- | --- | --- |
| Ensure participant use of cognitive skills | -Ensure that participants actually use the cognitive skills provided in the intervention in appropriate life settings. | -Use process measure; assess with questionnaire  -Use self-report regarding achievement of goals  -Use structured interview with participants  - Use exercises, goal sheets, and other printed material to foster adherence  -Assess mediating processes periodically  -Discuss ongoing use of skills with subjects  -Conduct follow-up discussions with participants |
| Ensure participant use of behavioral skills | -Ensure that participants actually use the behavioral skills provided in the intervention in appropriate life settings | -Assess with questionnaire  -Conduct self-report or self-monitoring and maintain activity log  -Measure objective of the intervention  -Maintain longitudinal contact (telephone) to encourage adherence  -Record time spent at facility  -Monitor frequency of sessions  -Use specific behavioral skill measure  -Follow up discussions with participants  -Conduct follow up discussions/telephone calls/counseling with participants |
